# Experimental Investigation of Microcontroller-Based Acoustic Temperature Transducer Systems

**DOI:** 10.3390/s23020884

**Published:** 2023-01-12

**Authors:** Ayman Y. Al-Rawashdeh, Tariq M. Younes, Ali Dalabeeh, Huthaifa Al_Issa, Mohamed Qawaqzeh, Oleksandr Miroshnyk, Andrii Kondratiev, Pavel Kučera, Václav Píštěk, Serhii Stepenko

**Affiliations:** 1 Department of Electrical Engineering, Faculty of Engineering Technology, Al Balqa Applied University, Al Salt 19117, Jordan; 2 Department of Mechatronics Engineering, Faculty of Engineering Technology, Al Balqa Applied University, Al Salt 19117, Jordan; 3 Department of Electrical and Electronics Engineering, Al Balqa Applied University, Al Salt 19117, Jordan; 4 Department of Electricity Supply and Energy Management, State Biotechnological University, 61052 Kharkiv, Ukraine; 5 Department of Materials Science and Engineering of Composite Structures, O.M. Beketov National University of Urban Economy in Kharkiv, Marshal Bazhanov Str. 17, 61002 Kharkiv, Ukraine; 6 Institute of Automotive Engineering, Brno University of Technology, Technická 2896/2, 616-69 Brno, Czech Republic; 7 Department of Electrical Engineering, Information and Measurement Technologies, Chernihiv Polytechnic National University, 14030 Chernihiv, Ukraine; 8 Research Institute of Electronics and Microsystem Technology, National Technical University of Ukraine “Igor Sikorsky Kyiv Polytechnic Institute”, 03056 Kyiv, Ukraine

**Keywords:** acoustic resonance, temperature measurement, standing wave, Arduino Uno

## Abstract

Temperature transducers are commonly used to monitor process parameters that are controlled by various types of industrial controllers. The purpose of this study is to design and model a simple microcontroller-based acoustic temperature transducer based on the variations of resonance conditions in a cylindrical resonance tube. The transducer’s operation is based on the generation of an acoustic standing wave in the free resonance mode of generation within a cylindrical resonance tube which is converted into a train of pulses using Schmitt trigger circuit. The frequency of the generated standing wave (i.e., the train of pulses) is measured by the Arduino Uno microcontroller, where a digital pin is used to acquire pulses that are counted using a build-in software function in an Arduino IDE environment. Experimental results are performed for three sizes of diameters to investigate the effect of the diameter of resonance tube on the obtained results. The maximum nonlinearity error according to Full-Scale Deflection (FSD) is about 2.3 percent, and the relative error of the transducer is evaluated using experimental findings and the regression model. The circuit simplicity and design of the suggested transducer, as well as the linearity of its measurements, are notable.

## 1. Introduction

In today’s world, temperature measuring is critical in a variety of industries. Temperature is an important feature of the state of matter because it reflects ongoing changes in the investigated process, such as changes in pressure and gas volume. Temperature is the most widely used and, in many cases, the most important characteristic in metallurgical, chemical, energy, and other industrial processes. Temperature measurements is necessary for a variety of physical, chemical, and biological processes [1]. Temperature measurement accuracy is critical for process automation and control [2]. As a result, in science and industry, precise temperature measurements and careful monitoring are required [3,4]. Various temperature sensors (or transducers) are utilized depending on the needed range and accuracy, including glass liquid thermometers, manometric thermometers, resistive sensing sensors, thermocouples, optical and photoelectric pyrometers, and acoustic temperature sensors [5]. The most frequent measurement methods rely on changes in material properties as a function of temperature. This change in material property is translated into an electrical parameter such as voltage, resistance, or current. Furthermore, a secondary measurement equipment records the electrical signals obtained by these procedures, or they are introduced into a signal conditioning circuit [6].

Acoustic temperature sensors and transducers have found applications in a variety of industries [7]; these transducers are mostly employed for measuring high temperatures [8] and the temperature of surrounding media, such as aqueous solutions [9]. The acoustic transducer is based on the idea that the velocity of sound changes as the temperature of the surrounding media changes (and thus the frequency). A temperature transducer is usually made of an acoustic transmitter and acoustic receiver. The transmitter produces a signal across the medium being tested, which is utilized to calculate the target temperature. The acoustic receiver receives the sent signal and converts it into an electrical signal. The acoustic signal receiver can be connected to sound collecting equipment, such as a sound card on a computer. The acoustic temperature sensor is made up of a variety of acoustic transmitters and receivers. A signal conditioner is required for these transmitters or receivers to function properly. The signal conditioner, on the other hand, can be a built-in device connected to the sound card or any other computer interface system. The computer functions as a signal processor, generating an acoustic signal with required parameters or processing an acquired signal to extract features such as frequency, amplitude, and phase. The retrieved features are obtained through the development of unique signal processing and data display methods.

The goal of this work is to design and model a simple microcontroller-based acoustic temperature transducer that uses a cylindrical tube to apply the resonance condition variation. Long-term stability, high sensitivity, and ease of interface circuit creation are the key advantages of acoustic transducers. The Arduino Uno microcontroller is used to create the suggested temperature measurement device. The circuit and design of the transducer are simple, resulting in higher measuring accuracy and sensitivity. The primary contributions of this paper are simplicity of design, low-cost data acquisition and signal processing technique, and acceptable nonlinearity of the acquired results.

Temperature transducers are commonly used to monitor process parameters that are controlled by various industrial controllers [10,11]. The conditions under which a temperature transducer is utilized are determined by several factors, including the transducer’s measurement range, accuracy, and speed response. The principle of conversion is the same regardless of the type of acoustic temperature transducer [12]. Specifically, signal conditioning circuits convert the measured temperature into an electrical signal. A resistive bridge, for example, is used to convert the change in resistance in a resistive temperature transducer into a corresponding voltage. After that, the output voltage is amplified and sent into an analog to digital converter or any other data acquisition system [13].

Temperature is also measured using a thermoelectric transducer [14]. A thermocouple’s operation is based on the thermoelectric effect, which states that in a closed circuit consisting of two dissimilar conductors, a thermo-emf (voltage) develops if the conductor junctions have different temperatures. Thermo-EMFs will arise on the connections of a closed loop made up of dissimilar conductors (thermoelectrodes), depending on the temperatures of the junctions *t* and *t*_0_ [15].

There are also acoustic temperature transducers [16,17,18,19] in addition to resistive temperature transducers and thermocouples. The acoustic transducer works on the idea that the speed of sound propagation in gases varies with temperature change. It is made up of an acoustic transmitter and acoustic receiver that sends and receives acoustic waves (spatially separated). An acoustic signal is generated by the transmitter and transmitted via the test medium. The speed of sound changes as temperature changes, and the receiver captures the acoustic signal for further processing.

The authors offer a temperature transducer based on sensing the frequency standing wave formed within a U-shaped resonance tube in their work [20]. The following is the transducer’s operation principle. In a glass tube filled with air, a standing wave is generated using a free resonance method of generation. The transmitter and the receiver are connected to the speaker input and MIC output of a personal computer sound card, respectively. The temperature of the media is calculated using LabVIEW software based on the measured frequency. The designed sensor has a sensitivity of roughly 0.476 Hz/°C, and the recorded temperature ranges from 20 to 120 °C.

In the work of [21], Acoustic sensors can act similarly, but because sound travels at such a slow speed, it cannot be measured directly from the moment the acoustic signal passes over a specified distance. A source that produces a sound signal, such as a speaker or an ultrasonic transmitter, is typically used in this type of sensor. The signal is conveyed through a tube filled with gas or liquid, with a microphone or another ultrasonic device (receiver) at the opposite end, and the measured temperature is between 50 and 130 degrees Celsius. The signal is transmitted, and the time difference between when it is sent and when it arrives at the receiver is calculated using a mathematical model, which is a formula that depends on the length of the tube and the time difference (flight time), resulting in the calculation of sound speed. After that, it may be calibrated to read temperature. The sensor’s sensitivity is around 50 Hz/°C, and the temperature measured ranges from 50 to 130 °C.

The authors in their paper [21] present a temperature transducer based on acoustic measurement. Acoustic resonance is also used to measure the temperature of the surrounding media. The transducer is made up of a sound speaker that generates standing waves in the forced mode inside a metallic tube with an open end. A thermoresistor, which is a katharometer detector, measures the amplitude of a standing wave. A resistive bridge connects the katharometer leads. The temperature change of the surrounding media is taken into account as the bridge’s output signal. The sensor’s sensitivity is around 0.20 mV/°C, and the temperature detected ranges from 10 to 100 °C.

The transducers presented in works [20,21] are constructed in a U-tube shape, which limits their application in practical applications. These transducers are connected to a computer via a sound card or a data acquisition system. The transducer given in studies [21,22] features a pricey temperature sensing component, namely a katharometer sensor, which is not suited for simple measurement. The suggested system features a sophisticated computer interface and unique equipment to perform the requisite temperature measurement in work [23]. As a result, it is difficult to use in simple applications.

In their paper [23], the authors describe a high-frequency temperature measurement system in which a transmitter generates a 15 kHz carrier signal modulated by a sinusoidal signal and an electric hydrophone receives the reflected signal. As compared to CTD (Conductivity–Temperature–Depth System Data), the system showed a 1.7 percent relative temperature error when compared to the reference system (Conductivity–Temperature–Depth System Data).

According to the studied literature, the developed sensors assume the following basic approach: a PC sound card is used to acquire the desired signal for processing, in addition to the need for signal conditioning circuits such as a bridge and amplifiers. Using the Arduino Uno microcontroller, this study seeks to establish the design and modeling of a basic, low-cost acoustic temperature transducer. The proposed transducer stands out for its circuit simplicity and design, as well as its measurement precision.

## 2. Materials and Methods

### 2.1. Theoretical Background

**Figure 1:**
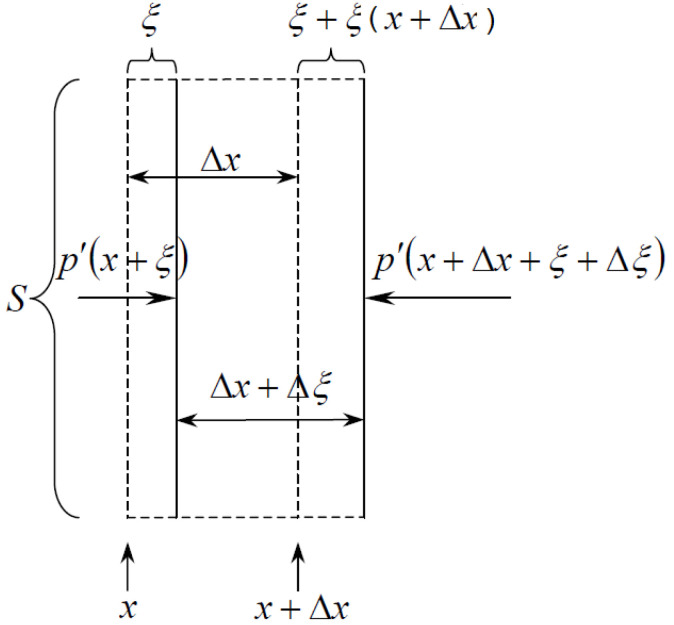
A volume of gas in the form of a cylinder with base area S and height Δx.

Sound is defined as mechanical (elastic) waves with frequencies ranging from 16 Hz to 20,000 Hz [24]. Let us consider the equation of oscillations of gas in a tube. Let us denote the coordinate of the particles located in some cross section of the pipe as *x*, and the displacement of the particles of this section along the axis of the tube during vibrations as *ξ*, then at the volume of gas in the form of a cylinder with a base area *S* and a height Δ*x* (Figure 1).

The mass of gas enclosed in this volume is equal to *ρS*Δ*x*, where *ρ* is the density of the gas at rest. If there are no oscillations of gas particles, then the pressure in the cross sections *x* and *x* + Δ*x* is the same and equal to *p* [25].

During oscillations, the displacements *ξ* of particles with different x at each moment of time turn out to be different. Therefore, the considered volume is deformed, and the pressure in different sections of the cylinder will no longer be the same. The instantaneous value of pressure in a certain section of the pipe can be represented as

(1) $$p^{\prime }=p+\Delta p,$$

where Δ*p*—the sound pressure.

Due to the smallness of Δ*x*, the acceleration projection on the *x* axis for all points of the cylinder can be considered the same and equal to $\frac{\partial^{2}\xi }{\partial t^{2}}$.

To find the projection onto the *x*-axis of the force acting on the volume under consideration, it is necessary to obtain the product of the tube base area *S* and the pressure difference *p*′ in the cross sections *x* + *ξ* and *x* + Δ*x* + *ξ* + Δ*ξ*:

(2) $$F_{x}=\left(p^{\prime }\left(x+\xi \right)-p^{\prime }\left(x+\Delta x+\xi +\Delta \xi \right)\right)S.$$

Expanding the instantaneous pressure *p*′ in a Taylor series around of the point *x*, neglecting the terms of higher orders, we obtain:

(3) $$F_{x}=S\left(p^{\prime }\left(x\right)+\xi \frac{\partial p^{\prime }}{\partial x}-p^{\prime }\left(x\right)-\left(\Delta x+\xi +\Delta \xi \right)\frac{\partial p^{\prime }}{\partial x}\right)=\;=-S\left(\Delta x+\xi \right)\frac{\partial p^{\prime }}{\partial x}\approx -S\Delta x\frac{\partial p^{\prime }}{\partial x}.$$

here we use the assumption Δ*x* >> Δ*ξ*.

Now we write down Newton’s second law for the allocated volume:

(4) $$\rho S\Delta x\frac{\partial^{2}\xi }{\partial \;t^{2}}=-S\Delta x\frac{\partial p^{\prime }}{\partial x}.$$

Reducing by *S*Δ*x*, we obtain the differential equation

(5) $$\rho \frac{\partial^{2}\xi }{\partial \;t^{2}}=-\frac{\partial p^{\prime }}{\partial x},$$

in which there are two unknown functions: *ξ* and *p*′. Let us express one of these functions in terms of the other. During fluctuations in the pipe, compression and rarefaction of the gas follow each other so often that adjacent sections of the medium do not have time to exchange heat, and the process can be considered adiabatic. In an adiabatic process, the relationship between pressure and volume of a gas is given by the equation $pV^{\gamma }=const$, where *γ* is the ratio of the heat capacity at constant pressure to the heat capacity at constant volume. According to this equation, we have

(6) $$p{\left(S\Delta x\right)}^{\gamma }=p^{\prime }{\left[S\left(\Delta x+\Delta \xi \right)\right]}^{\gamma }=p^{\prime }{\left[S\left(\Delta x+\frac{\partial \xi }{\partial x}\Delta x\right)\right]}^{\gamma }=p^{\prime }{\left(S\Delta x\right)}^{\gamma }{\left(1+\frac{\partial \xi }{\partial x}\right)}^{\gamma },$$

where *p*—the gas pressure at initial conditions.

We reduce by ${\left(S\Delta x\right)}^{\gamma }$ and expand the expression ${\left(1+\frac{\partial \xi }{\partial x}\right)}^{\gamma }$ in order by degrees $\frac{\partial \xi }{\partial x}$. Using the assumption that $\frac{\partial \xi }{\partial x}\ll 1$, and neglecting terms of higher orders of smallness. The result is the formula

(7) $$p=p^{\prime }\left(1+\gamma \frac{\partial \xi }{\partial x}\right).$$

We can express from it

(8) $$p^{\prime }=\frac{p}{\left(1+\gamma \frac{\partial \xi }{\partial x}\right)}\approx p\left(\left(1-\gamma \frac{\partial \xi }{\partial x}\right)\right).$$

From this expression we obtain

(9) $$\frac{p^{\prime }-p}{p}=-\gamma \frac{\partial \xi }{\partial x}.$$

Since *γ* is of the order of unity, then $\left|\frac{\partial \xi }{\partial x}\approx \frac{\Delta p}{p}\right|\ll 1$ that is, the deviation of pressure from the mean is much less than the pressure itself. This is true: for the loudest sounds, the amplitude of air pressure fluctuations does not exceed 1 mm Hg, while atmospheric pressure has a value of the order of 760 mm Hg.

Differentiating Equation (9) with respect to *x*, we find

(10) $$\frac{\partial p^{\prime }}{\partial x}=-\gamma p\frac{\partial^{2}\xi }{\partial \;x^{2}}.$$

Substituting this expression into Formula (5), we obtain the equation of displacement oscillations *ξ* in a tube with gas under the adiabatic law

(11) $$\frac{\partial^{2}\xi }{\partial \;t^{2}}=C^{2}\frac{\partial^{2}\xi }{\partial \;x^{2}}.$$

where *C* can be defined from continuous media mechanics that gives the following formula for the velocity of harmonic waves in a linear medium with elasticity to compression [25,26,27].

(12) $$C=\sqrt{\gamma \frac{R\theta \;}{\mu }}=k\sqrt{\theta },$$

where *R*—universal gas constant;

*μ*—molar mass;

*θ*—temperature in Kelvin; 

$k=\sqrt{\gamma \frac{R}{\mu }}$.

In theory, gas can be identified based on the magnitude of this ratio. We may compute the temperature by measuring the speed of sound in this method, assuming the molar mass of air is known and the specific heat ratio *γ* is equal to 1.4 for air [27].

### 2.2. Mathematical Model 

The standing wave method developed in free resonance mode [28,29] is used to determine the speed of sound (which is utilized to compute the temperature of the medium). Standing waves are generated by the superposition of two traveling waves propagating towards each other with the same frequencies and amplitudes. The occurrence of standing waves is a particular case of wave interference.

Consider the formation of a standing wave in a cylindrical tube with air closed at both ends. Let the wave incident along the positive direction of the *x*-axis be

(13) $$y^{\prime }\left(x,t\right)=A\cdot \sin\left[\omega \left(\tau -\frac{x}{C}\right)\right],\;\omega =2\pi /T=2\pi f=2\pi f/\lambda ,$$

where *y*—displacement (deviation from the equilibrium position) of the medium particle;

*x*—distance of the medium particle from the oscillation source;

*τ*—time;

*A*—amplitude (maximum displacement);

*ω* = 2π/T = 2πf = 2πf/λ—the cyclic frequency;

*T*—oscillation period, f is the oscillation frequency;

*C*—wave velocity;

*λ*—wavelength.

In this case, the oscillation phase changes in the opposite phase (by φ). Reflected wave $\xi_{2}\left(x,\tau \right)=A\cdot \sin\left[\omega \left(\tau +\frac{x}{C}\right)+\pi \right]$ propagating in the opposite direction of the *x*-axis interferes with the incident wave. Adding the equations of the incident and reflected waves, we obtain the standing wave equation:

(14) $$\xi =\xi_{1}\left(x,\tau \right)+\xi_{2}\left(x,\tau \right)=-2A\sin\omega \frac{x}{\upsilon }\cos\omega \tau .$$

Considering the condition for the resonance of vibrations in a pipe with air closed on both sides, the relationship between the length of the resonance tube and the wavelength is given by

(15) $$L=n\frac{\lambda }{2}.\;$$

where *L*—distance between the acoustic transmitter and the acoustic receiver.

*n*—integer number (for free resonance mode of acoustic wave generation *n* = 1).

The wave propagation velocity *C* is related to the wavelength *λ* and the oscillation frequency *f* by the relation, *C* = *λf*, whence, taking into account (15), for the first harmonic, we obtain

(16) C=2·L·f. 

Considering Equation (12), Equation (16) can be presented as

(17) $$C=\sqrt{\gamma \frac{R\theta \;}{\mu }}=k\sqrt{\theta }=2\cdot L\cdot f,\;$$

or we can write,

(18) $$\sqrt{\theta }=\frac{\left(2\cdot L\cdot f\right)}{k}.\;$$

To calculate the temperature in Celsius, the following formula can be used

(19) $$t=\frac{{\left(2\cdot L\cdot f\right)}^{2}}{k^{2}}-273.15.$$

Equation (19) shows that the temperature is affected by the length and the media that surrounds the speaker and the MIC in resonance condition. It is possible to compute temperature variations from the speed of propagation of a sound wave in a substance if the initial data are available.

Preliminary experiments were conducted to estimate the factors that significantly affect energy consumption. They were performed in accordance with the basic experimental research methodology [7].

By using the random balance method, the experiments were set up by the given design, and a number of significant factors were identified from the total number of factors and their interactions. Insignificant factors, such as hygroscopicity, looseness, interparticle space, and freshness of the grain, were suppressed. A full factorial experiment and a fractional factorial experiment were then conducted.

### 2.3. Transducer Design

**Figure 2:**
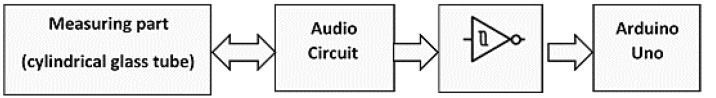
The block diagram of the transducer.

The acoustic temperature transducer is made up of a resonance tube filled by air (other gases may be used to improve the sensitivity of the transducer because of the effect of frequency obtained in resonance condition at fixed distance between the MIC and the speaker) with a cylindrical body that houses an acoustic wave receiver and transmitter. The tube has a linear shape, which makes it easier to immerse the transducer in a surrounding material than the transducers shown in works [20,21,22]. Furthermore, the transducer can be attached to any microcomputer system (or a microcontroller) without requiring the use of a sound card [30,31]. The transducer is shown in Figure 2 as a block diagram.

**Figure 3:**
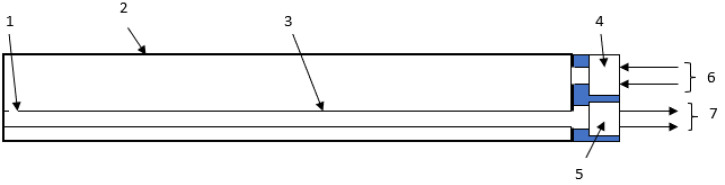
The sketch of the measuring part of the acoustic temperature transducer. 1—the open end of the inner tube, 2—the outer tube, 3—the inner tube, 4—the acoustic transmitter, 5—the acoustic receiver, 6—the lead wires of the acoustic transmitter, 7—the lead wires of the acoustic receiver.

**Figure 4:**
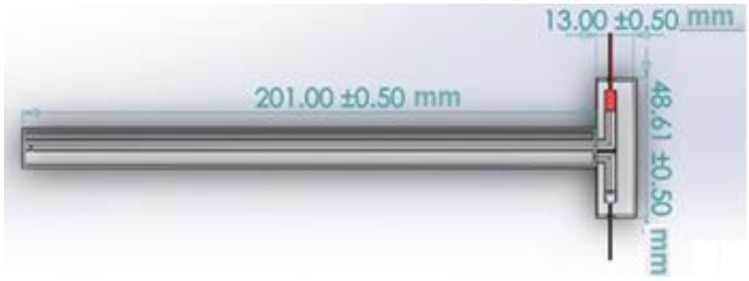
The design of the transducer utilizing Solidwork.

A cylindrical tube (external tube) with an internal diameter of about 12 mm and a length of about 200 mm made of glass serves as the measuring portion. Another cylindrical tube (internal tube, it is also made of glass) with a diameter of about 2 mm and a length of around 200 m is also included (a hole is located at the end of the inner tube). The transducer is made up of the following components. The acoustic signal transmitter is fixed on a cylindrical tube with a diameter of 12 mm, and the receiver is fixed on a cylindrical tube with a diameter of around 2 mm, and both are located at the same end of the tube. The internal cylindrical tube is located longwise to the outer cylindrical tube. Figure 3 depicts the transducer’s design using SolidWorks. Figure 4 depicts a sketch of the transducer’s measurement component.

It is vital to note that glass is not the ideal material for the sensor fabrication since its comparatively high thermal resistance which slows down the transducer’s dynamic response. Copper or another metal with lower thermal impedance might be a better choice.

The audio circuit is an electrical circuit that allows an acoustic receiver (a microphone) to receive an acoustic signal and amplifies it using an amplification circuit. 

**Figure 5:**
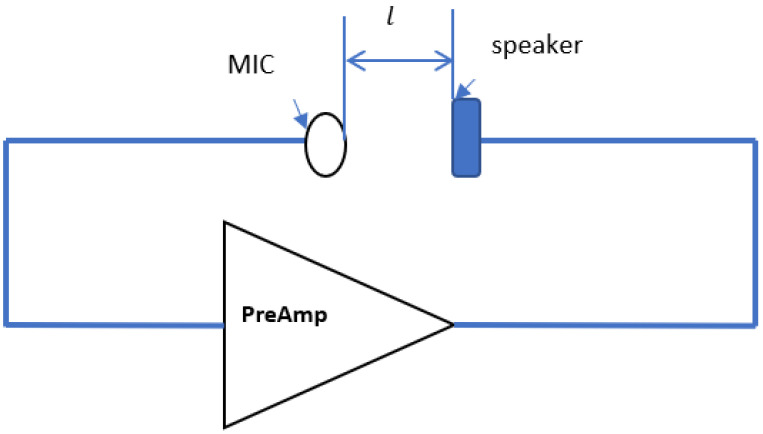
The block diagram of the resonance circuit.

The operation principle of the sound resonance which occurs in the audio circuit is based on the fact that when a mic and the speaker are connected to any electronic circuit and far from each other at distance l (Figure 5), the circuit develops resonance when it absorbs energy from an initial impulse and then continues to vibrate at the same frequency, but with decreasing amplitude, with no additional force acting against it. The resonant frequency of the system, denoted as *F*_0_, is the frequency at which this behavior manifests itself.

In the experimental setup, the acoustic transmitter is connected to the output of the amplified signal (speaker). For simplicity, the internal circuit of a “Handheld Karaoke Mic Wireless Microphone Bluetooth USB Speaker Player KT” is used as an audio circuit. The microphone is an electret condenser microphone with operating voltage about 5 Volts, the maximum current consumption is about 0.5 mA and internal impedance has a value less than 2.2 Ω, where the speaker is an earphone which has a diameter size of about 5 mm, with internal impedance about 16 Ω, and a sensitivity of about 103 db. 

The microphone leads are connected to a Schmitt trigger 74LS14, which reshapes the transmitter signal into a rectangular shape that Arduino pins can detect.

### 2.4. Operation Principle 

In this transducer, the acoustic resonance phenomena are used to measure temperature. Resonance is involved in a wide range of phenomena, some of which are beneficial and others which are harmful. Resonance is mostly employed as a helpful phenomenon in instrumentation and communication engineering. Electrical resonance allows the tuning of transmitters and receivers to certain frequencies and ensures that they operate without interfering with one another. The applications of resonant phenomena in electrical and radio engineering are numerous. Conservation laws, on the other hand, prohibit the use of resonance to gain free energy.

The operation principle of the transducer is based on the generation of an acoustic standing wave within a cylindrical resonance tube in the free resonance mode of generation [21]. The standing wave created within a tube is characterized by the length of the tube, the surrounding temperature, and the kind of gas in the tube. In the case of temperature transducer development, the length of the tube is fixed, and the tube is filled with air. Since the temperature is changed, the frequency of the created standing wave is changed. This frequency change represents the change in temperature in the surrounding media. 

The advantage of using the resonance mode is that the transducer parameters, such as tube length and media within the tube, are included in the measurement, thus eliminating the requirement for transducer calibration.

## 3. Results and Discussion

### 3.1. Simulation

**Figure 6:**
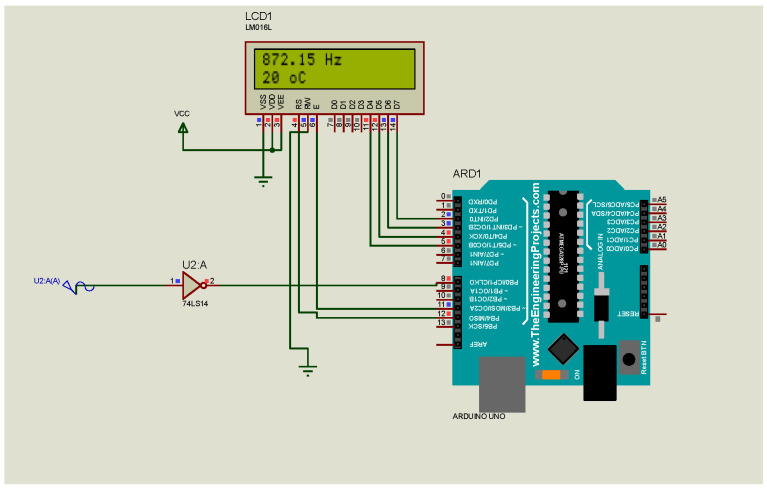
The simulation model of the acoustic temperature transducer.

The simulation model is developed using the Proteus software (Figure 6). The sound signal is replaced by the sine wave generator with a frequency that is generated at 20 °C. The output of the sound generator is connected to a Schmitt trigger 74LS14. The reshaped sinewave signal is introduced into PIN 8 of Arduino Uno. PIN 8 is a digital I/O pin that can be configured using Arduino IO functions. It accepts a digital signal as an input or produces an output signal as an output. Although the model works when the output of the microphone is directly connected to PIN 8, in experimental conditions, this test does not work. 

### 3.2. Software Development 

**Figure 7:**
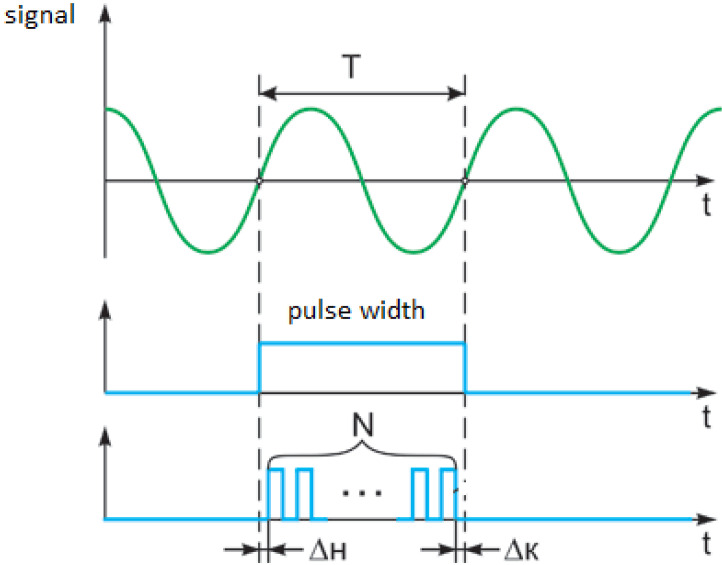
Pulse width method for measuring the period of a signal.

To calculate the temperature, we have to measure the frequency of the acoustic signal. This can be carried out by measuring the period *T* of generated signal (Figure 7). 

The frequency *f* is equal to the number of periods per unit of time. The frequency is associated with the period by a simple inverse ratio *f* = 1/*T*; therefore, by measuring the period, it is easy to calculate the inverse value—the frequency, and vice versa.

In principle, it is characterized by only one frequency *f*_0_ (and the corresponding period *T*_0_). The method of measuring the period is as follows. The comparator forms pulse width equal to the period *T*_0_. This pulse width is filled with pulses with a fixed stable frequency F (and period *t* = 1/*F*), the number of pulses *N* in the gate is counted. Then, the measured period will be equal to *T*_0_ = *N* × (1/*F*) = *N*/*F*, and the frequency *f*_0_ = *F*/*N*.

The software is developed using Arduino Uno, which has a special pulse function, which allows us to determine the duration of the positive or negative state of a rectangular signal. The pulse function measures the time during which a high or low logic level is present on PIN 8 of the Arduino Uno. Thus, in one signal period, we will have durations of positive and negative levels in microseconds. The pulse function measures time in microseconds. 

The software is developed using Arduino Uno, which has a particular pulse function, which allows us to determine the duration of the positive or negative state of a rectangular signal. The pulse function measures the time during which a high or low logic level is present on PIN 8 of the Arduino Uno. Thus, in one signal period, we will have durations of positive and negative levels in microseconds. The pulse function measures time in microseconds. The code for calculating temperature from the measured frequency is shown below:

1. #include <LiquidCrystal.h>
2. LiquidCrystal lcd(12, 11, 5, 4, 3, 2);
3. int Htime;
4. int Ltime;
5. float t;
6. float Gama = 1.4;
7. float R = 8.311472;
8. float M = 0.028;
9. float Ttime;
10. float f;
11. float L = 20E−2;
12. float k;
13. void setup ()
14. {
15.     pinMode(8, INPUT);
16.     lcd.begin(16, 2);
17.     k = sqrt(Gama * R / M);
18. }
19. void loop()
20. {
21.    lcd.clear();
22.    lcd.setCursor(0, 0);
23.    Htime = pulseIn(8, HIGH);
24.    Ltime = pulseIn(8, LOW);
25.    Ttime = Htime + Ltime;
26.    f = 1,000,000/Ttime;
27.    lcd.setCursor(0,0);
28.    lcd.print(f);
29.    lcd.print(“ Hz”);
30.    t = sq (2 ∗ L ∗ f)/sq(k) − 273.15;
31.    lcd.setCursor(0, 1);
32.    lcd.print(t);
33.    delay(500);
34. }

The program contains the following stages:

Stage 1: From line 1 to line 12;
  - Including LCD header file;
  - Declaration of required variables and constants;
Stage 2: From line 13 to line 18;
  - Defining Pin 8 as input;
  - Calling LCD function, which initializes the number of rows and columns;
  - Calculating the constant k;
Stage 3: From 19 to 34;
  - Configuration LCD;
  - Measuring the period;
  - Calculating the frequency;
  - Setting LCD cursor;
  - Calculating the temperature form;
  - Displaying the reading of frequency and temperature.

### 3.3. Experimental Setup and Experimental Results 

**Figure 8:**
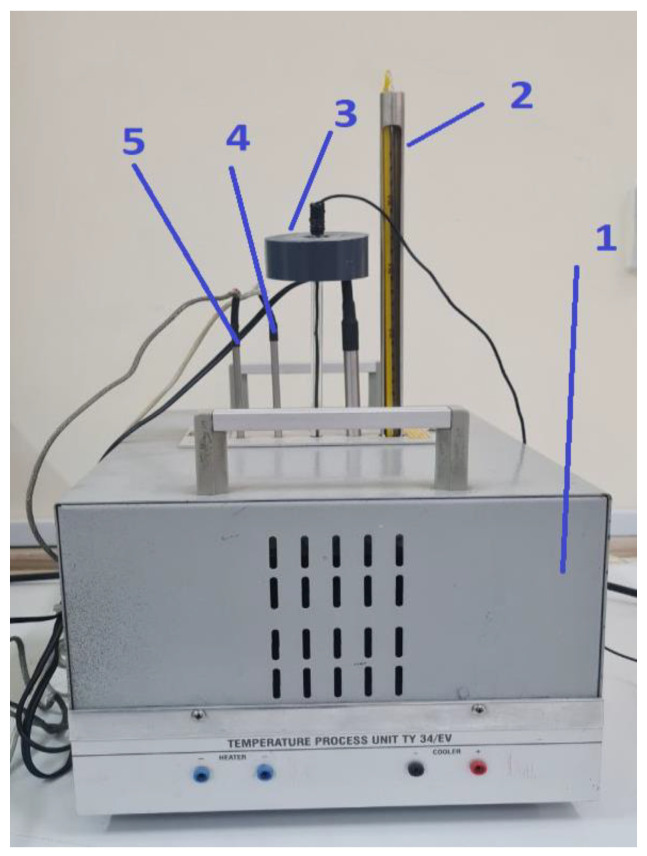
Heater system installation. (1—the heater system, 2—thermometer, 3—acoustic temperature sensor, 4—Pt100, 5 —NTC thermistor).

The experimental setup consists of the measuring part of the transducer placed in one of the heater’s housing systems, as shown in Figure 8. The usage of the heater system allows us to control the desired temperature range and distribute the heat in the sensor holders. 

The experimental procedure for measuring the temperature is the following:

1. The microphone output is connected to the Schmitt trigger input.
2. The output of the Schmitt trigger is connected to Arduino pin 8.
3. The cylindrical resonance tube (acoustic temperature sensor) is placed in the sensor holder 3.
4. The glass thermometer is placed in sensor holder 2.
5. Switch on the heater system and read the current temperature displayed on a glass thermometer.
6. Record the temperature of the glass thermometer and the measured frequency by Arduino Uno in Table 1.

**Figure 9:**
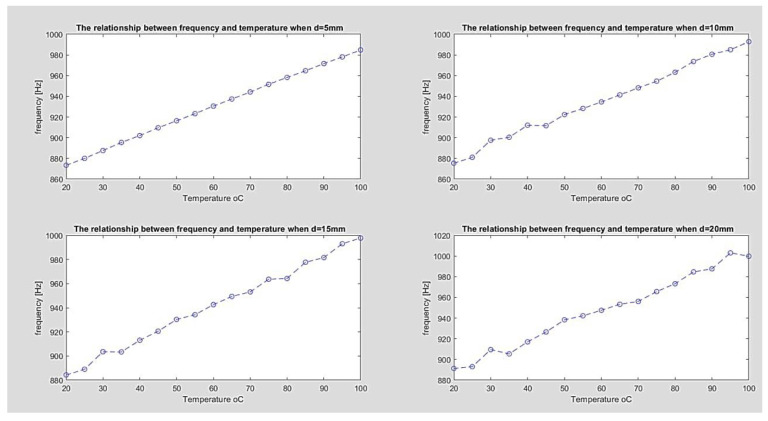
The relationship between frequency variation and temperature change for different diameters.

The relationship between frequency variation and temperature change is shown in Figure 9.

To represent the data, the method of data regression is used by utilizing Excel. Equation *y* = *ax* + *b* is used to fit the regression model of the transducer. It was found that the calibration curve has the following mathematical representation: *t* = 1.3934*f* + 846.62.

A linear regression model is utilized to create the calibration curve for relative error estimates; it was found that the highest relative error (according to FSD) is roughly 2.4 percent. Because the transducer tube can be formed of any insulation and wall thickness, environmental conditions such as humidity or vibration can have a substantial impact on transducer performance. The calculated R2 is around 0.9989, indicating that the chosen regression model is acceptable for fitting the excrement data set.

## 4. Conclusions

Using the Arduino Uno microcontroller, a simple, low-cost acoustic temperature transducer with a linear form has been designed and developed. The design is efficient and multipurpose. To perform the desired control action, the transducer could be integrated with any feedback of a process control system. The relationship between measured temperature and generated frequency was demonstrated to have a linear calibration curve.

Acoustic transducers provide several advantages, including long-term stability, high sensitivity, and ease of interface circuit design. The sensors that have been developed assume the following basic approach. In addition to signal conditioning circuits such as a bridge and amplifiers, a PC sound card is utilized to capture the desired signal for processing. The suggested transducer is notable for its circuit simplicity and design, as well as the accuracy of its measurements. Resonance mode has the advantage of including transducer parameters such as tube length and media within the tube in the measurement, which eliminates the need for transducer calibration.

## Figures and Tables

**Table 1:** Experimental results for temperature measurement using acoustic temperature sensor.

t_ref_, [°C]	f, [Hz]			
	d = 5 mm	d = 10 mm	d = 15 mm	d = 20 mm
20	873.31	875.31	884.31	891.31
25	880.01	881.01	889.01	893.01
30	887.57	897.57	903.57	909.57
35	895.38	900.38	903.38	905.38
40	902.06	912.06	913.06	917.06
45	909.60	911.60	920.60	926.60
50	916.35	922.35	930.35	938.35
55	923.24	928.24	934.24	942.24
60	930.57	934.57	942.57	947.57
65	937.39	941.39	949.39	953.39
70	944.16	948.16	953.16	956.16
75	951.65	954.65	963.65	965.65
80	958.27	963.27	964.27	973.27
85	964.71	973.71	977.71	984.71
90	971.64	980.64	981.64	987.64
95	978.07	985.07	993.07	1003.07
100	984.84	992.84	997.84	999.84

## Data Availability

The data presented in this study are available on request from the corresponding author.
